# Using a Co-design Approach to Create Tools to Facilitate Physical Activity in Children With Physical Disabilities

**DOI:** 10.3389/fresc.2021.707612

**Published:** 2021-11-15

**Authors:** Eline A. M. Bolster, Christa van Gessel, Maxime Welten, Sander Hermsen, Remko van der Lugt, Elles Kotte, Anita van Essen, Manon A. T. Bloemen

**Affiliations:** ^1^Research Group Lifestyle and Health, Utrecht University of Applied Sciences, Utrecht, Netherlands; ^2^Co-design Research Group, Utrecht University of Applied Sciences, Utrecht, Netherlands; ^3^Research Group Participation and Urban Development, Utrecht University of Applied Sciences, Utrecht, Netherlands; ^4^OnePlanet Research Center, Imec the Netherlands, Wageningen, Netherlands; ^5^Fitkids Foundation, Amsterdam, Netherlands

**Keywords:** tools, qualitative data, physical disabilities, children, co-design, physical activity

## Abstract

**Introduction:** There is a lack of effective interventions available for Pediatric Physical Therapists (PPTs) to promote a physically active lifestyle in children with physical disabilities. Participatory design methods (co-design) may be helpful in generating insights and developing intervention prototypes for facilitating a physically active lifestyle in children with physical disabilities (6–12 years).

**Materials and methods:** A multidisciplinary development team of designers, developers, and researchers engaged in a co-design process–together with parents, PPTs, and other relevant stakeholders (such as the Dutch Association of PPTs and care sports connectors). In this design process, the team developed prototypes for interventions during three co-creation sessions, four one-week design sprint, living-lab testing and two triangulation sessions. All available co-design data was structured and analyzed by three researchers independently resulting in themes for facilitating physical activity.

**Results:** The data rendered two specific outcomes, (1) knowledge cards containing the insights collected during the co-design process, and (2) eleven intervention prototypes. Based on the generated insights, the following factors seem important when facilitating a physically active lifestyle: a) stimulating self-efficacy; b) stimulating autonomy; c) focusing on possibilities; d) focusing on the needs of the individual child; e) collaborating with stakeholders; f) connecting with a child's environment; and g) meaningful goal setting.

**Conclusion:** This study shows how a co-design process can be successfully applied to generate insights and develop interventions in pediatric rehabilitation. The designed prototypes facilitate the incorporation of behavioral change techniques into pediatric rehabilitation and offer new opportunities to facilitate a physically active lifestyle in children with physical disabilities by PPTs. While promising, further studies should examine the feasibility and effectivity of these prototypes.

## Background

The benefits of encouraging a physically active lifestyle from an early age have been consistently documented (1). A meta-analysis showed an association between higher levels of physical activity and lower morbidity for typically developing children (2), and this is also assumed for children with physical disabilities. Moreover, physical activity is positively associated with psychosocial health for children with physical disabilities, including self-perceptions and health-related quality of life (3–5). However, most children, especially those with physical disabilities, do not meet the Dutch physical activity guidelines of at least 60 min of moderate-intensity activities every day (6–8).

While pediatric physical therapists (PPTs) are the designated professionals to facilitate physical activities in children with disabilities (9), a systematic review showed the lack of effective interventions available for PPTs to increase physical activity levels in these children (10). Increasing physical activity in children with disabilities is complex and multi-faceted, because of the variety of personal and environmental factors influencing physical activity (11–13). Important personal barriers are a lack of self-confidence, and feeling like an outsider (11). An important environmental barrier is the inability of people to see possibilities for children with disabilities to become physically active (11). Specific behavioral change strategies might provide PPTs with the right tools to support children and their parents to overcome personal and environmental barriers that hinder them to participate in physical activities (12, 14).

Appropriate opportunities at sports clubs and in the general community are important environmental requirements for children with disabilities to achieve a physically active lifestyle (11, 13). In the Netherlands, care sports connectors (CSC) aim to create opportunities for children with and without physical disabilities to become physically active, and in addition connect physical activity and sports professionals with health care sectors (15). Unfortunately, as yet, the collaboration between PPTs and CSCs has been lacking (16); sports participation in children might increase when collaborations between these two professions improve.

A disturbingly large part of interventions developed during scientific research are not used in clinical practice (17). This may be explained by a lack of attention for stakeholder acceptability and implementation in existing practices during the development of healthcare interventions (18). Actively engaging stakeholders, such as children, parents and healthcare providers, throughout all stages of intervention development could increase the actual use of healthcare interventions (19). Co-design, defined as collective creativity across the entire design process (20), is a design thinking methodology that has the potential to lead to the development of interventions that are more engaging, satisfactory, and useful to potential end-users. During co-design, an active collaboration occurs between researchers, designers, developers and end-users “as experts of their own experiences” (21), and, done rightly, co-design brings together their different views, input and competences (22). Knowledge transfer between stakeholders is important when developing and designing new interventions and co-design is characterized by incremental knowledge over time during a project (23, 24). Based on existing knowledge and generated insights, stakeholders can create principles for interventions. These principles can be transitioned into ideas and furthermore in testable prototypes. Because of its potential for improving implementation of newly developed interventions, co-design should be further examined in healthcare. Therefore, at first, examples of good practices are essential to increase knowledge about how co-design can be successfully applied in the development of interventions in pediatric rehabilitation (10).

This study presents a co-design approach for the development of prototypes containing behavioral change strategies for PPTs to facilitate physical activity in children with disabilities and a prototype to optimize collaboration between PPTs and CSCs. The first aim of this study is to describe the insights generated during co-design related to “facilitating physical activity.” The second aim is to describe the prototypes designed during co-design, based on knowledge from evidence and the generated insights during this method, to facilitate physical activity in everyday life settings of children with physical disabilities (6–12 years).

## Method

### Design

**Figure 1:**
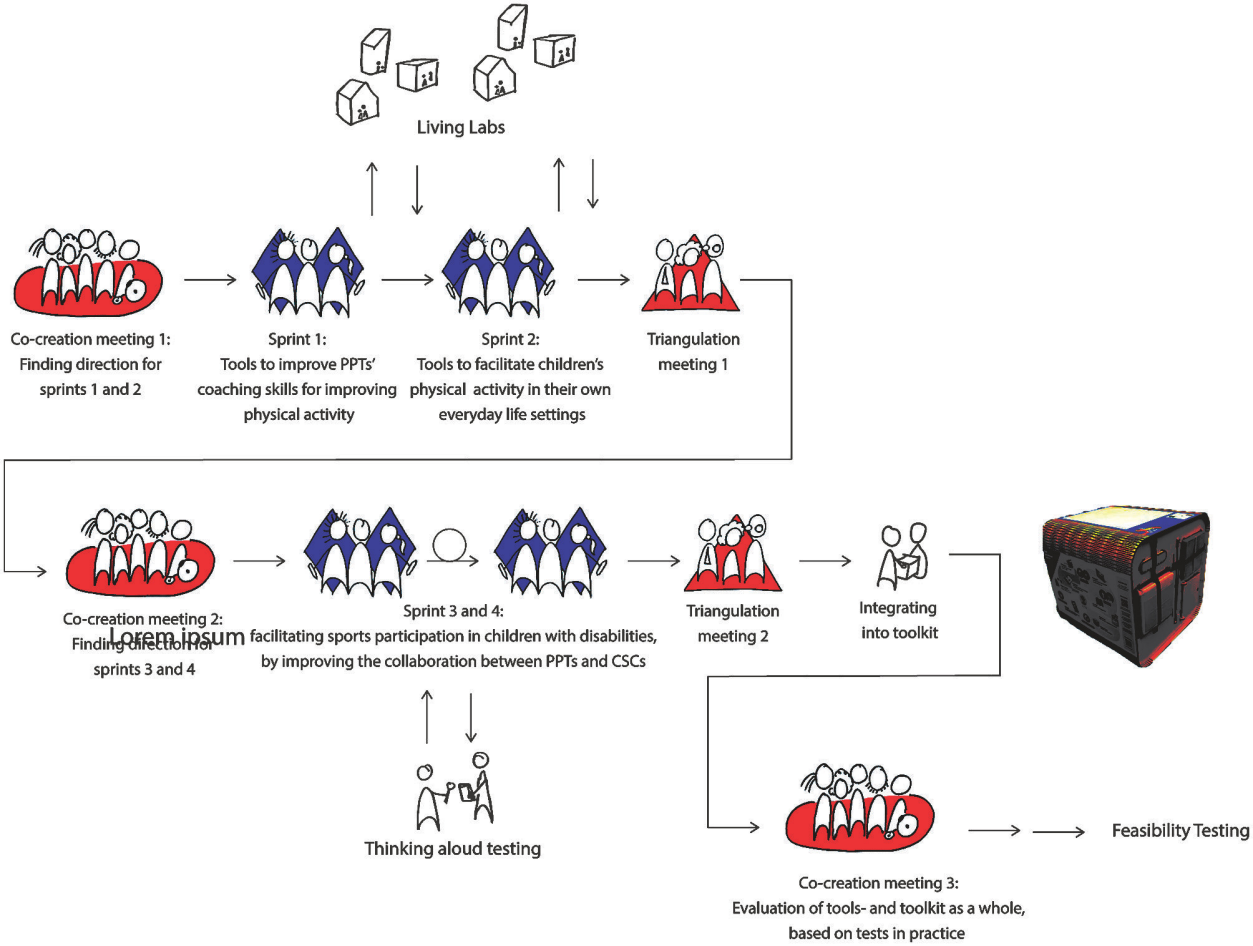
A visual representation of the co-design process containing three co-creation sessions, four one-week design sprints, living lab testing, and two triangulation sessions.

The present case study, called “What moves you?!” (funded by a grant from SIA, the Netherlands Taskforce for Applied Research, number RAAK.MKB08.006.), used different co-design methods to generated insights, and design and develop intervention prototypes. Collective decision-making and knowledge transfer between participating stakeholders was important during this process. Therefore, the principles of participatory action approach (PAR) were followed in this study. In PAR, stakeholder inclusion is extremely important and realized through active collaboration between stakeholders and researchers and there is a transfer of knowledge over multiple iterative development cycles (25), which is in accordance with co-design methods (26). Roughly, the co-design methods contained (1) three co-creation sessions, (2) four one-week design sprints (27–29) (3) living lab testing after each design sprint and (4) two triangulation sessions (Figure 1).

### Participants

Our consortium (*n* = 51) consisted of a broad range of stakeholders, such as parents of children with physical disabilities (*n* = 4), adults with a physical disability (*n* = 2), PPTs (*n* = 18), CSCs (*n* = 8), and others such as members from the Dutch parent association for children with a disability (*n* = 1), the Dutch Association of PPTs (*n* = 1), Fitkids Foundation (*n* = 2) which has the responsibility to ensure the quality of an exercise therapy program for children with a chronic condition or disability in the Netherlands; (30), the Knowledge Centre for Sports & Physical Activity Netherlands (*n* = 1), Special Heroes (*n* = 1) (Dutch organization promoting a healthy and active lifestyle for people with a disability), designers/developers (*n* = 2) and researchers (*n* = 12). A core team was responsible for planning, preparation, and execution of the co-design process including co-creation sessions, the four one-week design sprints, and the triangulation sessions. The core team of this project consisted of researchers with a background in PPT (*n* = 2), behavioral science (*n* = 2), design (*n* = 2), and social work (*n* = 1) and designers (*n* = 1). The consortium engaged in the co-creation sessions, furthermore PPTs and CSCs from our consortium tested the first prototypes of the tools in their own practice (living lab). Ethical approval for this study was granted by the healthcare ethics committee of the University of Applied Sciences Utrecht (99_000_2019).

### Co-creation Sessions

During the preparatory phase, the core team (*n* = 8) collected insights from literature and practice. The core team demonstrated these insights during co-creation sessions and evaluated if these insights resonated with the different stakeholders from the consortium, and if they could relate to these insights with their own (professional) experience. Furthermore, the core team used different generative techniques, such as mapping sessions, during co-creation sessions to evoke tacit knowledge and latent needs (21). *Mapping* is a method to create a visual representation of interacting variables to facilitate the understanding of complex systems (21). These methods were used to explore the ideas, needs and values from stakeholders beyond their first response by collecting, for example, their wishes, dreams and barriers for potential interventions. By using generative methods, the core team empowered a large variety of stakeholders to participate during co-creation sessions; this stimulated and improved knowledge transfer, with an increase of insights over time.

After the first co-creation session, the core team defined the focus for the sprints based on knowledge from evidence and insights generated during this session. During the first sprint, the team focused on the development of prototypes to improve PPT's coaching skills for improving physical activity. During the second sprint, the team focused on the development of prototypes to facilitate children's physical activity in their own everyday life settings. During the third and fourth sprint, the core team focused on facilitating sports participation in children with disabilities, by improving the collaboration between PPTs and CSCs.

### Sprint Weeks

**Figure 2:**
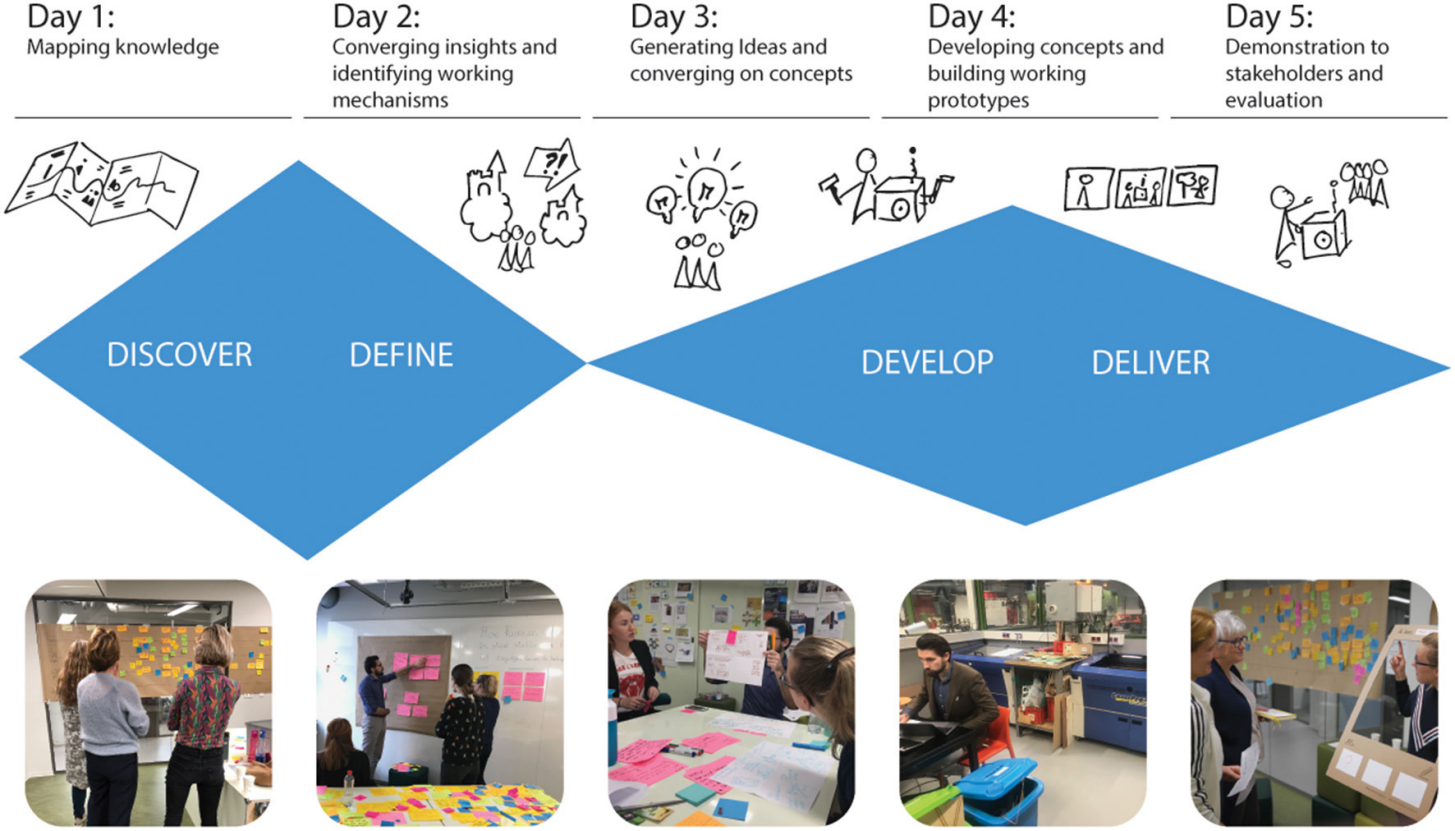
A visual representation of a 5 day sprint week.

To design the prototypes, the core team (*n* = 8) set up four design sprint weeks based on the Google Design Sprint approach (28). This approach consists of a 5 day process for answering critical development questions through design, prototyping, and testing ideas with stakeholders. The goal of each sprint was to quickly develop feasible prototypes for interventions based on knowledge from evidence, generated insights during this project and user testing, with maximum attention to stakeholder participation. We used the adopted Double Diamand described by Elbers et al. (19) for the sprint weeks containing two sequences of diverging and converging. See for a visual representation of the sprint week (Figure 2).

On the first sprint day, the core team went through a divergent phase in which they collected and mapped available knowledge from literature, practice and co-creation sessions with different co-design methods such as context mapping sessions, user journeys and socionas. The *user journeys* enable stakeholders to collaboratively construct a timeline that illustrates the journey of a child with physical disabilities and a goal related to increase physical activity from the start of PPT treatment (31). *Socionas* are a tool to stimulate designers to incorporate the systemic view around a child into the design process (32). A sociona consists of a visual representation of the dynamics in a system of people (for example children, parents, PPTs, CSCs on micro level and stakeholders setups on macro level) (32). After collating the insights, the core team performed further user research on day one with stakeholders (involving 4–8 stakeholders depending on the sprint week) from the consortium for instance by performing in-depth interviews with stakeholders. Stakeholders also had the opportunity to reflect on the collected data.

The second sprint day focused on converging activities, by selecting emergent themes from the insights gathered on day one. Based on these themes we determined the main working mechanism driving the behavior change intended by the interventions. For example one of the targeted working mechanisms was “support children in creating their own solutions.” The other targeted working mechanisms are described in Figure 2. Determining such working mechanisms is important in prototype development, because they give insights in the expected efficacy of the intervention.

On the third sprint day, using different brainstorming techniques, the core team went through a divergent phase, by generating ideas for prototypes fitting these main working mechanisms. The team used the Behavorial Lenses Approach to integrate insights on individual determinants of behavior in the design activities (33). At the end of day 3 we focused on converging activities by selecting the concepts for the most promising prototypes using guiding principles for the interventions developed on the first sprint day.

On day 4, the designers of the core team (*n* = 3) developed working versions of the prototypes to make the working principles tangible (34). Each sprint finished on day 5, with a demonstration lunch in which the core team presented the prototypes to stakeholders from the consortium and colleagues. After the demonstration lunch, prototypes were adjusted based on their feedback. A reflective session, in which the core sprint team evaluated the sprint week, took place at the end of each sprint week.

### Living Lab Testing

At the end of sprint week 1 and 2, the prototypes were sent to fourteen PPTs to allow them to interact with the prototypes in their daily practice (34). They tested these prototypes together with children with physical disabilities and their parents. To reflect on these prototypes, structured telephone interviews with the PPTs were conducted after 4 weeks. During sprint three and four, the team designed (sprint 3) and developed (sprint 4) a mobile app to improve collaboration between PPTs and CSCs. This application was tested in a structured environment by children with physical disabilities, their parents, PPTs and CSCs to allow these participants to interact with this prototype. During these structured tests the participants were encouraged to “think aloud;” two or three observers documented this feedback.

### Triangulation

To validate the prototypes, the core team organized triangulation sessions. Two behavioral scientists and two experts in social dynamic systems, all unrelated to the project, reviewed the prototypes, and especially their underlying working mechanisms. The behavioral scientists focused on the integration of behavioral insights in the prototypes. They identified which Behavioral Lens(es) they observed in the mechanism of the prototypes (33). The experts in social dynamic systems pointed out where prototypes responded to social aspects of behavioral change. All findings were then discussed by these experts and the core team.

### Qualitative Analyzes

Three researchers with a background in PPT, design, and social work, independently structured and analyzed all available data from the “What moves you?!” study. The data consisted of sprint reports, reflective journals (daily self-reports in which the core team collected their experiences and thoughts on the co-design process) (35), and photos and film clips of co-creation sessions, sprint activities and triangulation sessions. Using Atlas.ti, we used a qualitative method to analyze our co-design data (36). An inductive thematic approach was used in which we coded fragments of text in step one, resulting in subthemes in step two (37, 38). In step 3, finally, we determined main themes. Step one consisted of defining a text or visual section as an important insight obtained during the co-design process. These insights should help answer three questions used to develop interventions during the four sprints (1) to improve PPT's physical activity coaching, (2) to facilitate children's physical activity in their own life settings, and (3) to improve collaboration between PPTs and CSCs in order to facilitate sports participation. Consensus between the three researchers was reached throughout this entire process. These themes, subthemes and quotes are also gathered in knowledge cards. Rather than solely disseminate knowledge among researchers through scientific articles we created these knowledge cards to ensure that the gathered insights from this study will reach and be used by PPTs.

After testing the prototypes in the living lab settings, the interviews conducted with PPTs (*n* = 16) were recorded and summarized. A content analysis was performed to determine barriers and facilitators in the usability of the tools. These barriers and facilitators were used to optimize the prototype of the tools.

## Results

At the end of this process there were two specific outcomes (1) generated insights collected during co-design, and (2) prototypes of the tools.

### Insights

**Figure 3:**
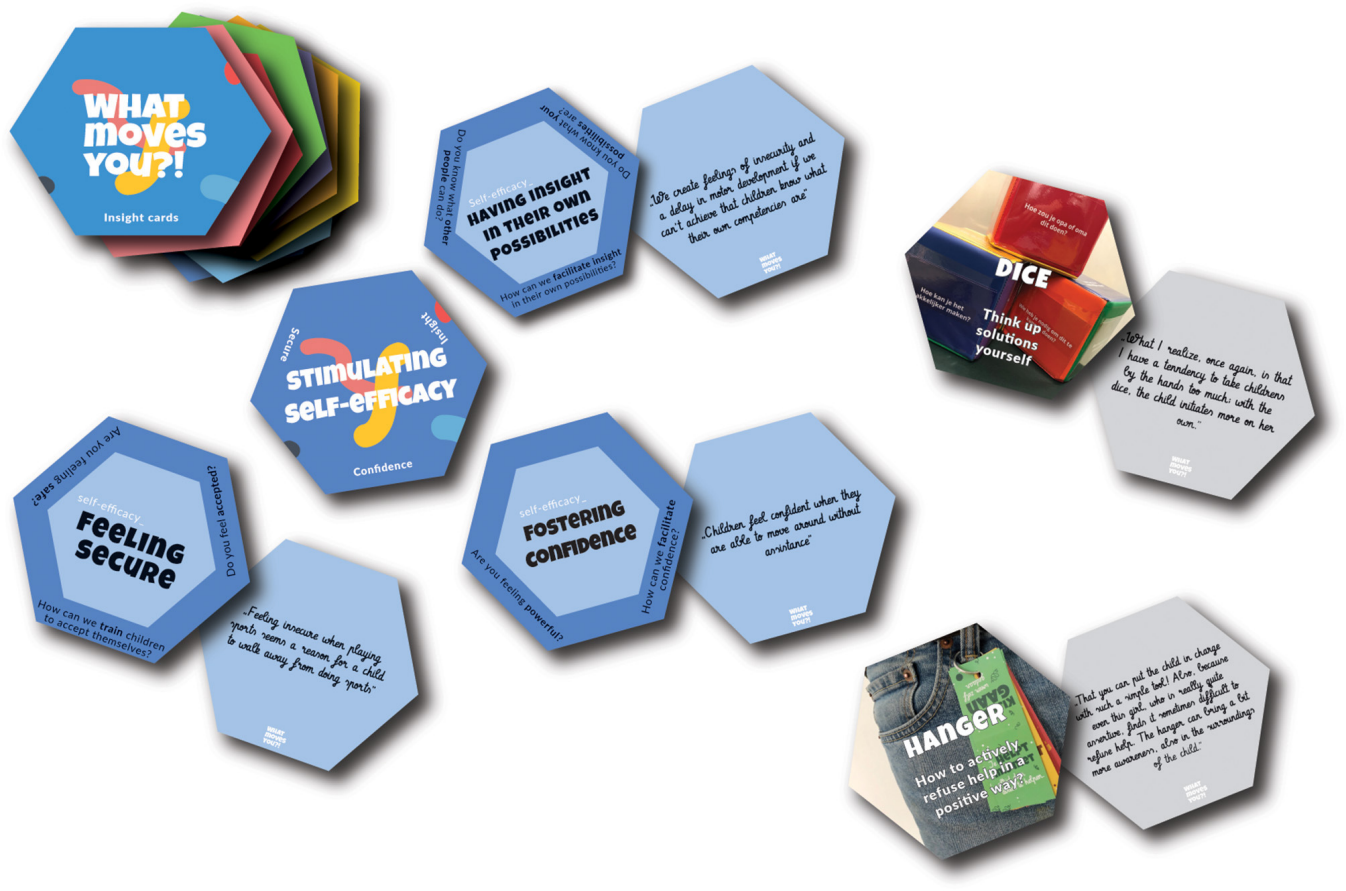
Example of knowledge cards with the theme *stimulating self-efficacy*.

Table 1 presents an overview of the themes and subthemes gathered from all co-design data and the most important issues related to these themes and subthemes are discussed in the text below. The themes are included in the headings and both the themes and subthemes are in *italic* in the text below. The quotes represent the summarized translation of what parents, adults with a physical disability, PPTs, CSCs, researchers and other stakeholders expressed during this co-design approach. Because of the intensive collaboration during this process it was not documented who was the author of the quote. Figure 3 shows an example of a knowledge card with the theme *stimulating self-efficacy* and the subthemes *fostering confidence, fostering feeling secure* and *having insight in their own possibilities*.

**Table 1:** Themes and subthemes.

**Stimulating self-efficacy**	**Stimulating autonomy**	**Focusing on possibilities**	**Focusing on the needs of the individual child**	**Collaborating with stakeholders**	**Connecting with a child's environment**	**Meaningful goal setting**
Fostering confidence	Being able to deny help	Focusing on abilities instead of obstacles	Using a tailored approach	Striving for equality	Have activities take place in daily life	Relevant goals
Fostering feeling secure	Knowing who is responsible	Creative solutions	Finding suitable solutions	Finding the right support	Have activities take place in a meaningful environment	Purposeful goals
Having insight in their own possibilities	Knowing their own boundaries	Having fun	Giving the child a central position	Sharing knowledge	Including the social environment	Goals focusing on participation
Being motivated	Being able to create their own solution	Challenging solutions	Listening to each other	Monitoring the child	Fostering visibility	
	Being able to try out activities	Small steps toward goal				
	Trial and error	Celebrating (actual) successes				

#### Stimulating Self-Efficacy

For *stimulating self-efficacy, fostering confidence, fostering feeling secure, having insight in their own possibilities* and *being motivated* were pointed out as positive subthemes. Positive experiences for *fostering confidence* were described when children were able to move without assistance, for example when self-propelling their wheelchair instead of being pushed. *Fostering feeling secure* seems important when performing a sports, as mentioned by a CSC: “Feeling insecure when playing sports seems a reason for a child to walk away from a sports.” *Having insight in their own possibilities* was pointed out as important when facilitating a physically active lifestyle: “We (parents, PPTs and CSCs) create feelings of insecurity and a delay in motor development if we can't achieve that children know what their own competences are.”

#### Stimulating Autonomy

The importance of *stimulating autonomy* for children with a physical disability was mentioned often. Participants believed that, in order to become autonomous, it is important for children are able *to deny help, know their own boundaries* and *know who is responsible*. Where possible, children with a physical disability need the opportunity *to deny help*. However, parents and healthcare providers are often over-protective and over-supportive, and they often provide help immediately; as mentioned by a parent: “I find it hard to give my child the opportunity to deny help and become independent.” Healthcare providers expressed the need to discuss *who is responsible* for achieving formulated therapy-goals: “it is important that I know where my responsibility stops and where the responsibility of a child, their parents or other healthcare providers starts.” It is furthermore important for a child to *know what their boundaries are* when the goal is to become more physically active and autonomous: “by doing and discovering a child will experience their boundaries. So, dare to search for their real boundaries.”

After becoming autonomous, the importance of staying autonomous was highlighted. A solution-oriented approach, where children are able *to create their own solutions*, seems a positive factor for being autonomous; as mentioned by a PPT: “I withdraw to see if the child comes up with his own solution,” “I would rather act too late than too early to increase their exploratory behavior.” This was also pointed out by an adult with a physical disability: “Often healthcare providers take control. For example, bus drivers often push children in a wheelchair to the school bus, even if these children are able to self-propel their wheelchair. What are the consequences of this behavior for a child's psyche?” The need *to try out new activities* was also mentioned: “By doing and discovering a child will discover their own limits.” A solution-oriented approach is characterized by *trial and error*: “You should give children the opportunity to make mistakes, but without judgment if things go wrong.”

#### Focusing on Possibilities

Focusing on *abilities instead of focusing on obstacles* is essential when stimulating a physically active lifestyle: “ask what a child can do instead of what they can't do.” The way to achieve this is to create solutions in a child's own environment that are *creative, fun* and also *challenging*. The importance of *small steps* toward the final goal was highlighted in order to *celebrate (actual) successes*.

#### Focusing on the Needs of the Individual Child

When a (health) care professional is focused on facilitating a physically active lifestyle, it is important to *focus on the needs of the individual child* and their parents. The provided care must therefore have an *tailored approach* and the solutions for increasing physical activities must be *suitable* for a child and their environment: “Healthcare providers should sense what a child needs,” “It involves customization, while protocols do not take the real needs of a child into account.” The *child must have a central position* when providing care: “often we talk about children when we have to talk with children.” This is only possible when stakeholders actually are *listening to each other* as mentioned by parents of a children with physical disabilities: “I wish professionals would really listen to parents in an open conversation without prejudice caused by the diagnosis,” “It feels like fighting when I'm not heard.”

#### Collaborating With Stakeholders

The importance of *collaborating with stakeholders* was commonly reported when discussing how adequate sports activities can be found for a child. Within the Netherlands CSCs and PPTs have the opportunity to collaborate when searching for sports, together with children and their parents. During this collaboration it is important that conditions are created in which all stakeholders *feel equal*. *Sharing knowledge* is one of the key ingredients to strive for equality: “The PPT probably knows better what the possibilities of a child are, but the CSC often knows more about relevant sports activities.” *Finding the right support* for a task is often challenging for children and their parents and also for healthcare providers: “It is difficult to find the right healthcare provider who can guide the child toward a sports.” As mentioned before, children with a disability often walk away from sports. Therefore, *monitoring the child* when starting and playing sports is important “dropping out from a sports might also be good, a child has tried and we now know that this does not work.”

#### Connecting With a Child's Environment

*Connecting with a child's environment* was often mentioned when discussing how to facilitate children's physical activity in their own life settings. First of all, interventions should focus on being active in *daily life* situations; as explained by a PPT: “You try to provoke the child to move differently in their own environment.” Therefore, PPTs must leave their own practice and include the *meaningful environment* of a child in their routine: “A success factor is going outside, into a child's own environment,” “during the treatment” and “at home” are two different worlds.” *Including the social environment* is another key ingredient when facilitating a physically active lifestyle: “Involving parents is not just letting parents watch, but let them participate and experience,” “Friends of a child sometimes come to my treatment so that these children can learn skills together and integrate this activity at home (for example when playing tag).” If a child wants to *connect with their environment* it is important that a child is *visible*, so that, for example, other children in their own environment know who they are. This was explained by an adult with a physical disability: “It is important to make yourself visible to other children in your own environment. The older you get, the more difficult this is.”

#### Meaningful Goal Setting

*Meaningful goal setting* was often mentioned as one of the most important aspects of a healthcare intervention. For children, parents and their healthcare providers it is important that goals are *relevant* and *purposeful* and that the main goal of the therapy is focused on *facilitating participation*. When goals are *relevant* this will motivate children and their parents to achieve their goals: “it is important to set goals for the intervention together with children and their parents.” Goals should be *purposeful*: “it is important that goals are clear for children and their parents and not vague.” Furthermore, because increased participation in physical activities should be the main focus of an intervention, goals should ideally be set on “participation” level; “the main goal should focus on participation,” “during an intervention, don't solely focus on activities such as walking, but focus on participation, for example moving from one place to another.”

### Prototypes of the Tools

Eleven tool prototypes were designed during the sprint weeks (Table 2): four physical tools to improve PPT's physical activity coaching, four physical tools and two information videos to facilitate children's physical activity in their own life settings, and a mobile app to improve collaboration between PPTs and CSCs.

**Table 2:** The designed tools including pictures, the targeted working mechanism and a description of the tools.

**Prototype**	**Picture**	**Targeted working mechanism**	**Description**
My Diary to improve PPTs' physical activity coaching	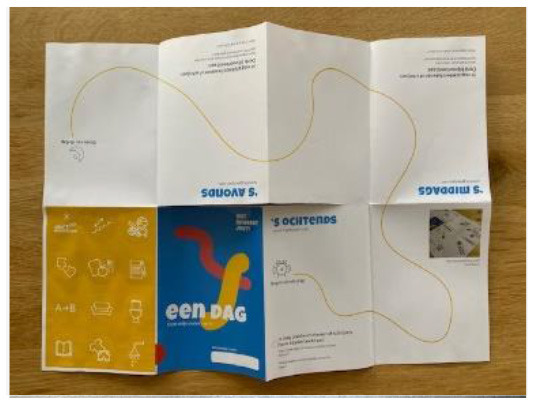	Integrate the social and meaningful everyday context of children in a PPT's treatment.	A diary for a child and their parents (separately) to track the amount of help a child is getting / parents are giving during a single day. This diary can be discussed during a PPT's session with a child and their parents to create awareness of the existing habits.
Look through the Window to improve PPTs' physical activity coaching	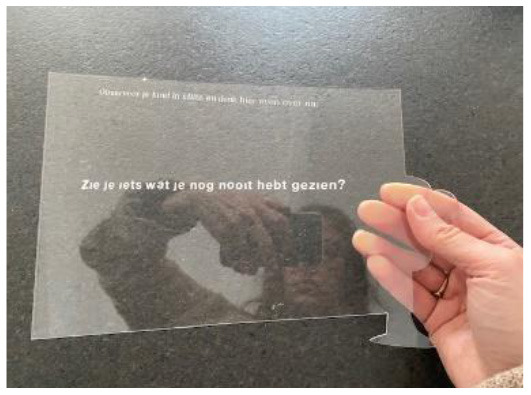	Facilitate a positive, observing role for parents during a PPTs treatment.	Parents can hold this window during a PPTs sessions. Instead of interfering in a conversation and/or intervention, they are invited to observe their child and discuss their findings afterwards. All questions encrypted in the window are positively formulated.
Question Dice to improve PPTs' physical activity coaching	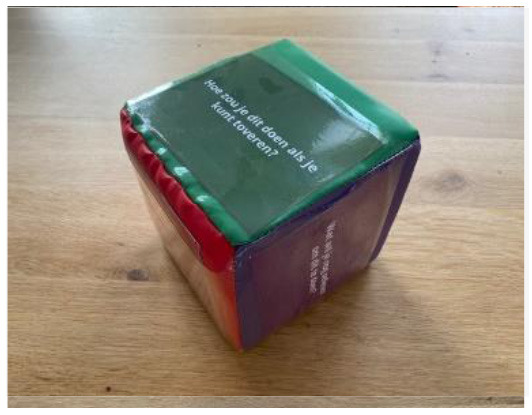	Support children in creating their own solutions.	These question dice help a child create and try their one solution. After rolling the dice, the child is confronted with a question that stimulates a creative solution, e.g., “how would your superhero achieve this?”
Fears, Dreams, Actions Card set to improve PPT's physical activity coaching	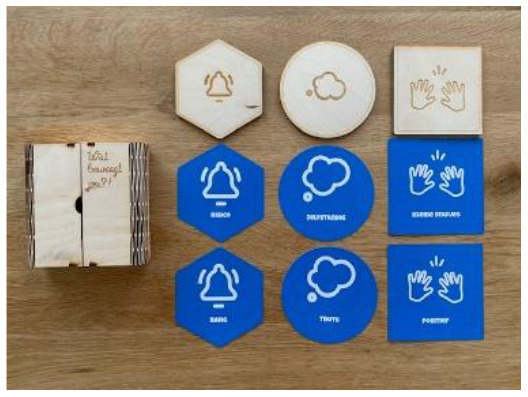	Support parents in releasing their child.	This card set helps to discuss the fears and obstacles that a child and/or their parents might have when setting a meaningful goal. After discussing their fears, a child's and parent's dreams are discussed. Based on these dreams, the PPT, parent and child can formulate actions to achieve their goals.
Conversation placemat to facilitate children's physical activity in their own life settings	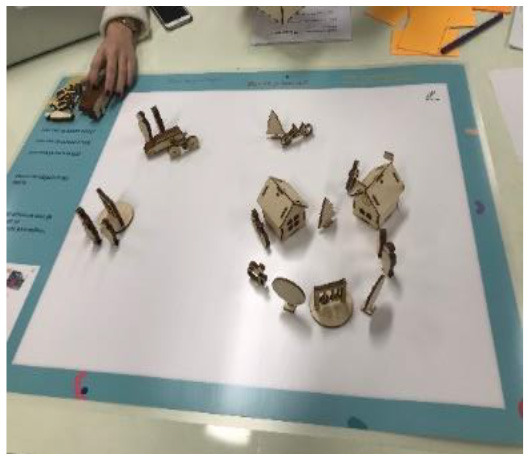	Provide insight into a child's opportunities and obstacles in their own environment.	This placemat helps to create a better understanding of the social and physical environment of a child. 3D figures (persons, houses, trees, cars, wheelchairs, etc.), can be placed on the placemat and a child can write or draw on the placemat. The child, their parents and the PPT can create a visual overview of the child's environment. Together with child and parents, the PPT can discuss opportunities and obstacles in a child's own environment.
Key ring to facilitate children's physical activity in their own life settings	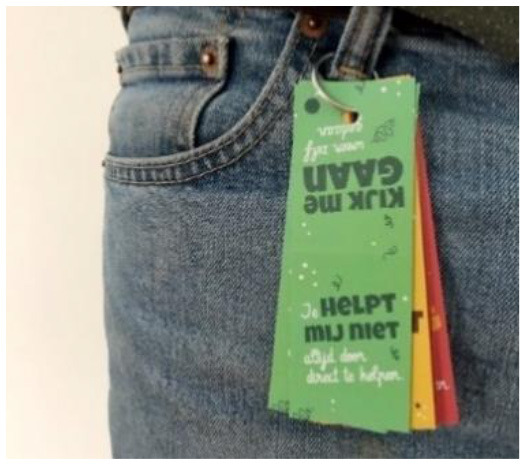	Help a child refuse unwanted aid actively.	A child can attach this key ring to their clothes, backpack, or any other spot. Different labels are attached with messages such as “look at me, I did it myself!” and “you won't help me by helping without asking”. The child can pull of a part of the label and present this to the person who wanted to help without asking.
Stickers to facilitate children's physical activity in their own life settings	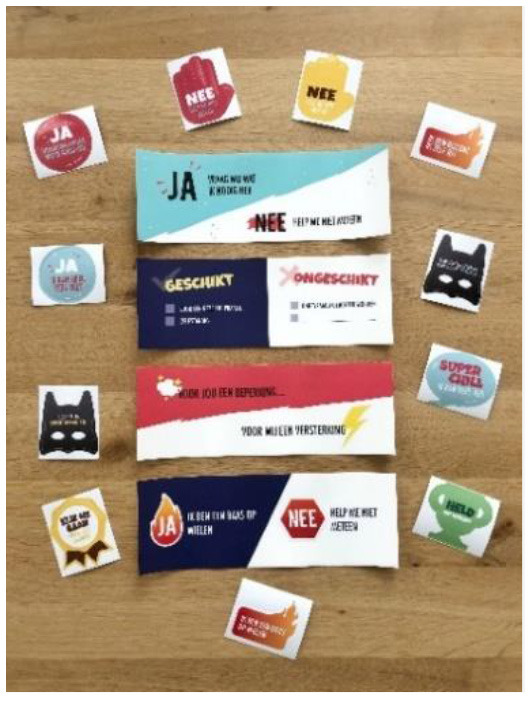	Help a child refuse unwanted aid passively.	The stickers have messages like “yes, ask me what I need,” and “I'm my own superhero”. The stickers can be placed on a wheelchair, backpack, clothes etc. The stickers have a creative design and the messages are positively formulated.
Clapboard to facilitate children's physical activity in their own life settings	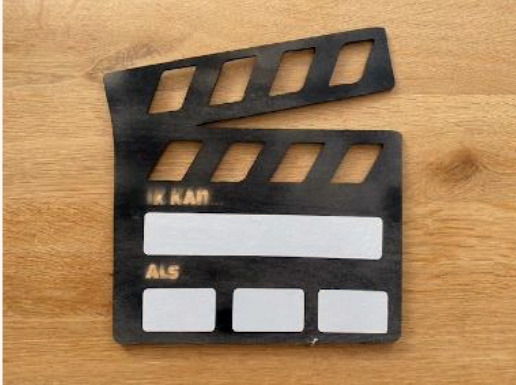	Improve the clinical handover between healthcare providers with a specific role for a child and his parents.	The video frame gives a child and his parents the opportunity to present their own goals of a healthcare intervention to healthcare providers with a video. Or to show, with a video, what a child is capable of. This improves the handover between e.g., PPTs and doctors, or PPTs and teachers.
Information video's to facilitate children's physical activity in their own life settings	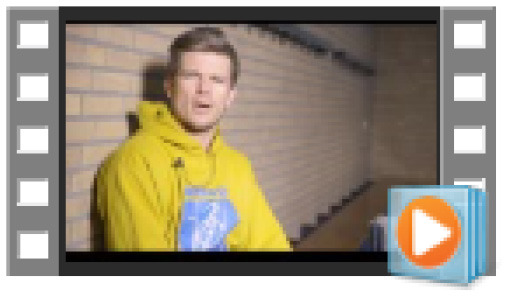	Inform children, parents and healthcare providers about the positive effects of an active lifestyle.	The videos discuss the effects of stimulating self-efficacy by, for example, refusing unwanted help and the importance of connecting with the environment. Both videos are created by adults with a physical disability.
Application what moves us? to improve collaboration between PPTs and CSC	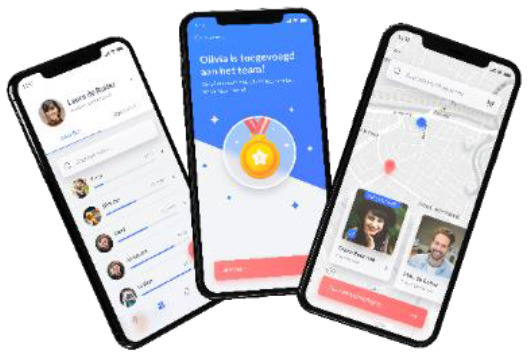	Improve collaboration between PPTs and CSCs in order to facilitate sports participation for children.	The application makes it possible for PPTs and CSCs to search for PPTs and/or CSCs in their community (through a google map overlay). They can link a sports professional to a child, and they can track the progress of a child when searching and performing a sports.

## Discussion

This study shows how a co-design approach can be successfully applied to generate insights and develop interventions in pediatric rehabilitation. The study had two aims: firstly, to describe all insights on how PPTs, CSCs, parents, and others can support children with physical disabilities in active lifestyles, obtained during the co-design process. The second aim was to describe the interventions designed in the co-design process. Regarding the first aim we found the following positive factors of importance for children, their parents and (health)care professionals: (1) stimulating self-efficacy, (2) stimulating autonomy, (3) focusing on possibilities, (4) focusing on the needs of the individual child, (5) collaborating with stakeholders, (6) connecting with a child's own environment, and (7) meaningful goal setting. Regarding the second aim, to describe the designed intervention prototypes based on evidence and generated insights from the co-design process: these designed prototypes focus on determinants of behavior (i.e., self-efficacy and autonomy), possibilities, and connecting with a child's own environment. These intervention offer new opportunities to PPTs and CSCs to support children with physical disabilities in obtaining a more physically active lifestyle.

The generated insights during this co-design process reflect the importance of creating interventions aiming at behavior for facilitating physical activities in children with physical disabilities; as yet, hardly any such interventions exist (10). The shift from improving functions (i.e., physical fitness) and activities (i.e., motor skills) toward supporting determinants of behavior by PPTs was also underlined in a recent study of Reedman et al. (14). When facilitating a physically active lifestyle, an individually tailored approach, focusing on the needs of the individual child (11, 14), and setting meaningful goals (14, 39–41) are often mentioned in the literature (as well as in this study), however, the shift toward behavioral support is rather new in pediatric rehabilitation. Reedman et al. concluded that clinicians should, for example, focus on optimizing motivation and stimulating self-efficacy (14). Stimulating self-efficacy, by increasing confidence, security, and motivation, was also mentioned by many of our stakeholders–parents, adults with physical disabilities and PPTs. Furthermore, stimulating autonomy was often mentioned during our co-design activities. To become and stay autonomous, it is important that a child gets to know their own boundaries, that they are able to deny help, and that they can create their own solutions rather than adopting the solutions provided by parents or healthcare professionals. In sum, (health) care providers, such as PPTs, should focus on supporting determinants of health behavior when facilitating a physically active lifestyle.

One of the main competences of a PPT is creating fun and playful interventions for children. Having fun while being active in daily life activities and sports is very important for increasing leisure-time activities. As a consequence, having fun might increase physical activity levels (42). Stakeholders in this study underlined that focusing on abilities rather than obstacles is important for a PPTs intervention and that having fun, being creative and celebrating (actual) successes should be integrated in their interventions. However, PPTs should make a shift from creating a fun environment in a PPT's session to creating fun in everyday physical activities (43). Connecting with the everyday environment, and integrating the meaningful and social environment in their interventions, was mentioned as important and difficult. Darrah et al. (44) created a context approach were therapists are trained in changing tasks and environmental factors rather than changing the abilities of a child. When using this context approach it is important that the interventions take place in the natural environment of a child, while PPTs interventions are still mostly taking place in their own practices.

Stakeholders mentioned the importance of supporting behavior, focusing on possibilities and providing therapy in the natural environment of a child is important, as confirmed by literature (43). However, PPTs in this project also mentioned a lack of knowledge and tools to focus on these elements during their interventions. Furthermore, literature shows that logistics, time and (as a consequence) costs make it difficult to provide therapy in the everyday environment of a child (44). Rather than solely disseminate knowledge among researchers through scientific articles we created knowledge cards to ensure that the gathered insights from this study will reach and be used by PPTs. Furthermore, we designed and developed tools focusing on behavior and connecting with the environment. While the generated insights during this co-design approach provides directions for a PPTs intervention, we have not yet evaluated the efficacy of the designed tools. Therefore, the next step is to combine these tools in one toolbox and conduct a feasibility study and then an effectiveness study, to examine whether this toolbox actually increases PPTs efficacy to facilitate physical activity in children with physical disabilities.

One of the strengths of a co-design approach is the possibility to include many stakeholders with different backgrounds, as done in this study. However, co-design is time consuming (45), and capturing and documenting the knowledge transfer during co-design is difficult because the amount of data and the different sorts of data (e.g., interviews, photos of mapping sessions). During this project one researcher was responsible for collected all available data and therefore the knowledge was captured and documented carefully and, while structuring and analyzing the data was time consuming, the generated insights during this project provide a wide overview of expert knowledge related to the theme “facilitating physical activity.” However, because of the active collaboration between different stakeholders the author of a quote is not documented. Therefore, the data does not represent separate views from parents, adults with a physical disability and professionals.

## Conclusion

A co-design approach is an effective way to generate insights and explore new interventions for healthcare providers such as PPTs and CSCs. They can benefit from this co-design approach because it affords a better understanding of their needs. The designed prototypes facilitate the incorporation of behavioral change techniques into pediatric rehabilitation and thereby offer new opportunities to facilitate a physically active lifestyle in children with physical disabilities. Our findings suggest that when facilitating a physically active lifestyle, it is important to focus on (1) stimulating self-efficacy, (2) stimulating autonomy, (3) focusing on possibilities, (4) focusing on the needs of the individual child, (5) collaborating with stakeholders, (6) connecting with a child's own environment, and (7) meaningful goal setting.

## Data Availability Statement

The raw data supporting the conclusions of this article will be made available by the authors, without undue reservation.

## Ethics Statement

Written informed consent was obtained from the consortium partners for the publication of any potentially identifiable images or data included in this article.

## Author Contributions

EB, CG, MW, SH, RL, EK, AE, and MB were all responsible for data collection. EB, CG, and MW performed the qualitative data analyses with MB as supervisor. EB and MB were primarily responsible for writing the article but all other authors contributed also. SH, RL, and MB contributed to the study design.

## Funding

Parts of this research were funded by SIA, the Netherlands Taskforce for Applied Research No. RAAK.MKB08.006. One Planet research Center was funded by the Province of Gelderland.

## Conflict of Interest

The authors declare that the research was conducted in the absence of any commercial or financial relationships that could be construed as a potential conflict of interest.

## Publisher's Note

All claims expressed in this article are solely those of the authors and do not necessarily represent those of their affiliated organizations, or those of the publisher, the editors and the reviewers. Any product that may be evaluated in this article, or claim that may be made by its manufacturer, is not guaranteed or endorsed by the publisher.
